# The perception of emotional cues by children in artificial background noise

**DOI:** 10.1007/s10772-020-09675-1

**Published:** 2020-01-22

**Authors:** Emilia Parada-Cabaleiro, Anton Batliner, Alice Baird, Björn Schuller

**Affiliations:** 1 Chair of Embedded Intelligence for Health Care and Wellbeing, University of Augsburg, Eichleitnerstr. 30, F2, 86159 Augsburg, Germany; 2 GLAM - Group on Language, Audio & Music, Imperial College London, London, UK

**Keywords:** Emotion perception, Noise, Developmental age, Cross-cultural, Nonsense speech, Paralinguistics, Multi-modality

## Abstract

Most typically developed individuals have the ability to perceive emotions encoded in speech; yet, factors such as age or environmental conditions can restrict this inherent skill. Noise pollution and multimedia over-stimulation are common components of contemporary society, and have shown to particularly impair a child’s interpersonal skills. Assessing the influence of such features on the perception of emotion over different developmental stages will advance child-related research. The presented work evaluates how background noise and emotionally connoted visual stimuli affect a child’s perception of emotional speech. A total of 109 subjects from Spain and Germany (4–14 years) evaluated 20 multi-modal instances of nonsense emotional speech, under several environmental and visual conditions. A control group of 17 Spanish adults performed the same perception test. Results suggest that visual stimulation, gender, and the two sub-cultures with different language background do not influence a child’s perception; yet, background noise does compromise their ability to correctly identify emotion in speech—a phenomenon that seems to decrease with age.

## Introduction

As the acquisition of affective skills runs parallel to cognitive developmental stages (Piaget 1962), emotional understanding also increases over the first stages of life (Friend and Bryant 2000). In the development of such emotional awareness, vocal communication has a crucial role, being one of the first channels to be acquired and developed for perceiving and expressing emotion (Darwin 1872). Yet, noise pollution, an unavoidable factor of modern life, has shown to have a noticeable effect on children (Fritschi et al. 2011). For listeners with normal hearing, this might recreate a condition typical of those with hearing-impairments: The ability of hearing-impaired users to perceive emotions from speech has shown to be reduced both for children (Öster and Risberg 1986; Most and Michaelis 2012) and adults (House 2009). Furthermore, the digital era has led to auditory and visual over-stimulation that became a typical feature of everyday-life, especially affecting a child’s interpersonal skills (Klorer 2009). Despite this, the impact of multi-modal stimuli on children’s perception of emotional speech has received minimal attention (Shackman and Pollak 2005); how noise pollution affects normal-hearing adults’ perception of emotion has been investigated only recently (Parada-Cabaleiro et al. 2017; Scharenborg et al. 2018), and children’s perception in background noise has only been assessed for the linguistic understanding (Bent and Holt 2018), while the paralinguistic component of the message has not been evaluated so far.

For evaluating the influence of noise on the perception of emotional speech, we chose pink noise to artificially recreate adverse environmental conditions—this noise type was found to particularly affect a listener’s ability to correctly identify emotions in speech (Parada-Cabaleiro et al. 2017)—as well as two emotionally connoted drawings of a tree (Winston et al. 1995) in order to recreate multi-modal (audio + visual) affective stimuli, that is, emotional speech (audio) and emotional drawings (visual) in concomitance. Since culture might influence children’s perception of emotional speech (McCluskey and Albas 1981; Matsumoto and Kishimoto 1983), we considered children from two (sub-)cultures with different language backgrounds, namely Spanish and German. Following research focused on children’s vocal expression of emotion (Batliner et al. 2008), this work encourages the understanding of children’s perception of emotional speech. In turn, it might inspire interdisciplinary research with a focus on childhood, for example, in order to develop technology-based tools for educational purposes. Such applications would be in line with those oriented towards promoting children’s emotional intelligence through the utilisation of virtual reality (Finkelstein et al. 2009).

The rest of the manuscript is laid out as follows: Sect. 2 sketches the state of the art in the field; Sects. 3 and 4 present methodology and statistical design, Sects. 5 and 6 results and discussion; Sect. 7 details limitations of the presented work; finally, future goals and conclusions are given in Sect. 8.

## State of the art

The theory of cognitive development (Piaget 2000) states that a child’s acquisition of knowledge is a learning process divided into four stages: *Sensorimotor* (0–2 years; in this stage, the acquisition of new knowledge is based on sensory and motor experiences, such as the manipulation of physical objects); *Preoperational* (2–7 years; a stage characterised by the beginning of symbolic thought and the acquisition of language; still children tend to be mostly egocentric, that is, they have difficulties in understanding other’s perspective); *Concrete Operational* (7–11 years; in this stage, the inductive logical reasoning applied to concrete events is developed); and *Formal Operational* (from 11 years upwards; a stage in which the deductive logic emerges and children start to reflect about concepts that require abstract reasoning, such as moral or philosophical problems); cf. Table 1 for the distribution of our subjects group. Children’s perception of emotional speech has been studied extensively (Friend and Bryant 2000; Morton et al. 2003; Waxer and Morton 2011; Quam and Swingley 2012). Despite this, and even though the ability to perceive emotions from non-verbal cues is progressively acquired during the second (preoperational) developmental stage (Friend and Bryant 2000; Morton et al. 2003), children’s perception of multi-modal emotional cues, that is, of emotional information encoded in different channels simultaneously (e. g., audio–visual), has been addressed rarely (Shackman and Pollak 2005)—and never, to the best of our knowledge, in the earlier stages of development.

As children can have difficulties in understanding the concept of emotional dimensions (Russell 1980), such as arousal (related to the intensity of the emotion) or valence (related to the positive or negative hedonistic value), most studies that evaluate children’s perception of emotion employ a categorical model (Morton et al. 2003; Waxer and Morton 2011; Shackman and Pollak 2005; Quam and Swingley 2012) where every emotional state is defined by a unique category (Scherer 1984). Furthermore, since perception studies can be specially tedious for children, for whom sustained periods of attention may be difficult (Gumenyuk et al. 2001), strategies as evaluating a reduced number of stimuli (Matsumoto and Kishimoto 1983) through interactive computer-based tasks (Morton et al. 2003) guarantee more reliable results.

## Methodology

The tendency of a child to prioritise some cues belonging to another modality, such as facial expressions or linguistic content, over the vocal/non-verbal message encoded, for example, in voice quality, could depend on his/her cognitive elaboration of the instructions instead of an inability to perceive paralinguistic information (Morton et al. 2003). This could be the reason why linguistic meaning (Morton et al. 2003) or cross-culturally accepted emotional icons such as facial expressions (Shackman and Pollak 2005) may bias a child’s perception of emotion in multi-modal stimuli. Thus, we chose stimuli void of emotional linguistic meaning, that is, nonsense speech, and a visual input with non-standardised emotional connotation.

### Stimuli

Since linguistic meaning influences both adults’ (Friend and Farrar 1994) and children’s perception of emotional speech (Morton et al. 2003), the linguistic content has been obscured in previous research by considering a foreign language and pass-filtering the samples (Friend and Farrar 1994; Morton and Trehub 2001). Nonsense utterances, commonly used in cross-cultural studies to highlight the non-verbal emotional component, have been extensively used for assessing adults’ perception (Scherer et al. 2001)—but rarely for children (Matsumoto and Kishimoto 1983).

We thus chose the nonsense acted emotional utterance *ne kal ibam soud molen!*, pronounced in the three emotional states happiness,^1^ anger,^2^ and sadness, from the *GEneva Multimodal Emotion Portrayals* (GEMEP) database (Bänziger et al. 2006), used in the ComParE 2013 challenge (Schuller et al. 2013). The considered nonsense utterance was created to represent a plausible pseudo-linguistic phoneme sequence with a similar pronunciation in several Western languages (Bänziger and Scherer 2010). Due to this, it is specially suited to test our two groups of listeners (Spanish and German) who could perceive it as a pronounceable foreign language (Scherer et al. 2001); the sentence was produced by a native French actress, i. e., a ‘foreigner’ with respect to both language and sub-culture. In addition, unlike previous research that created nonsense utterances for each cultural sub-group (Matsumoto and Kishimoto 1983), by that we guarantee identical conditions by using the same nonsense stimulus. According to previous research on children’s perception of emotional speech (Matsumoto and Kishimoto 1983; Morton and Trehub 2001), to avoid the influence of gender, for this study, we used only a female speaker.

**Fig. 1:**
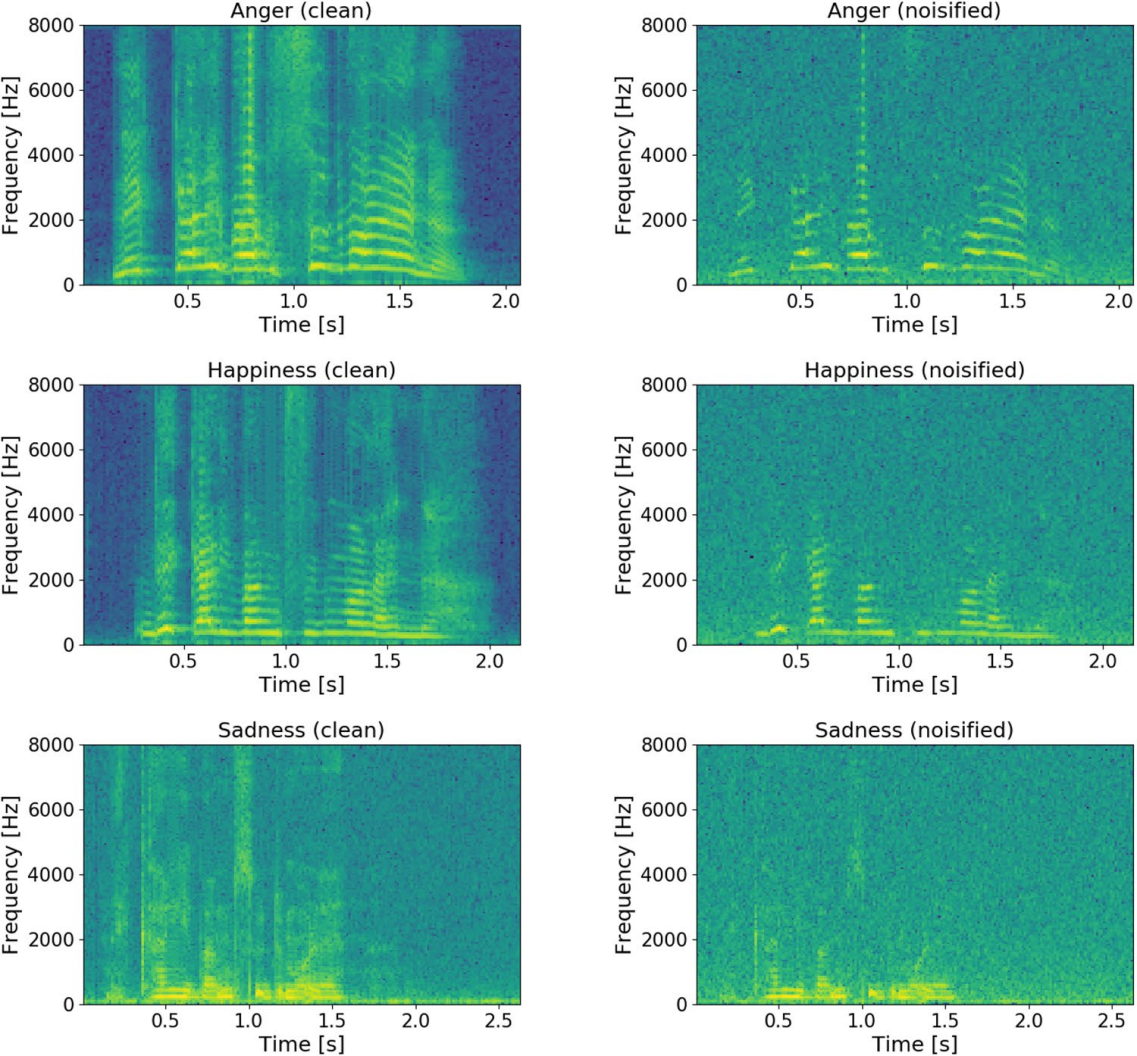
Spectrograms of the clean (left) and noisified (right) nonsense emotional utterances: anger, happiness, and sadness (from top to bottom), produced by a female speaker; x-axis: duration in sec.; y-axis: frequency between 0 and 8 kHz

We employed an artificially polluted background created in *Matlab R2014a* (Mathworks 2014) through the addition of *pink noise* at a Signal-to-Noise Ratio (SNR) of $-1$ dB; this was found—due to its higher intensity in the low frequency band when compared with brownian and white noise—to be especially effective in reducing listeners’ ability to correctly identify emotions in speech (Parada-Cabaleiro et al. 2017). For the audio stimuli, we considered six utterances: three clean (i. e., without the addition of background noise) and three noisified. In Fig. 1, a comparison between the clean and the noisified samples’ spectrum is given, showing that pitch modulation and spectral variance, which are relevant features for emotion recognition, are specially affected in the noisified samples.

**Fig. 2:**
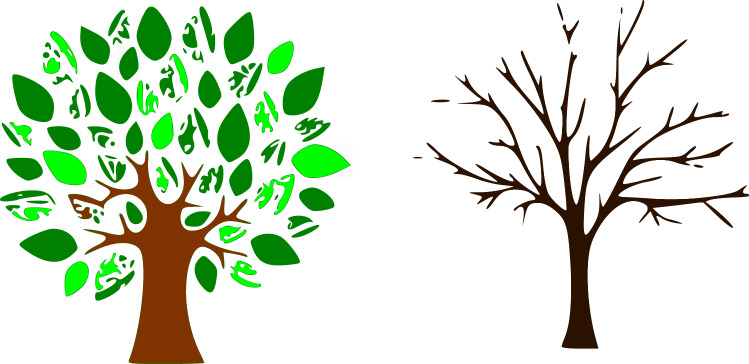
The emotionally connoted drawings used as visual stimuli in the presented study: positive (left), and negative (right)

Children express and perceive emotion encoded in drawings from the age of three years (Misailidi and Bonoti 2008). Drawings of trees have been extensively investigated, those with leaves, green, and robust, being identified as positive, and thin ones without leaves as negative (Ives 1984; Winston et al. 1995). Two emotionally connoted drawings (taken from an online stock-image library and modified by the authors) were chosen for the visual stimuli: a ‘positive’ and a ‘negative’ tree (cf. Fig. 2). These were evaluated by the children without audio, in order to guarantee the intended emotional connotations. From the 109 children, the positive drawing was correctly identified by 100 (five misclassified it with sadness and four with anger); the negative one was correctly perceived by 99 children (88 identifying it with sadness, 11 with anger, and 10 misclassified it as happiness). Since the children who misclassified the visual stimuli did not belong to a specific age group, and their responses did not differ from those given by others, we did not exclude their responses. Moreover, Sect. 5 shows that the visual stimuli seem not to influence the children’s perception of emotional speech.

### Test design

**Fig. 3:**
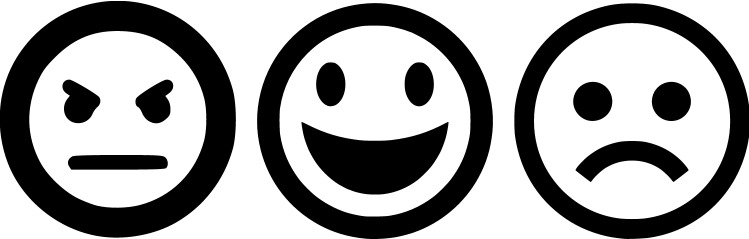
The emotionally expressive images of faces used in our study, representing each of the three forced-choice categorical test responses: anger, happiness, and sadness (from left to right)

We evaluated the perception of anger, happiness, and sadness—emotions which are commonly studied to evaluate a child’s perception of emotional speech (Shackman and Pollak 2005; Matsumoto and Kishimoto 1983). A forced-choice categorical computer-based perception test, hosted on the on-line platform *Typeform*,^3^ was conducted. Three drawings of emotional facial expressions (displayed in Fig. 3), representing the three possible test responses anger, happiness, and sadness, were used as reference, because they are more adequate than verbal labels for a perceptual assessment of children (Waxer and Morton 2011; Matsumoto and Kishimoto 1983).

Each child assessed 20 emotionally connoted stimuli: 8 mono-modal (6 audio and 2 visual) and 12 multi-modal (audio + visual). The 6 audio stimuli, lasting 2. 1, 2. 2, and 2. 6 s for anger, happiness, and sadness, respectively, are the three emotional utterances, both clean and noisified. The 2 visual stimuli are the positive and negative drawings.^4^ The 12 multi-modal stimuli are obtained by the combination of the previous, that is, the 6 audio and the 2 visual stimuli presented concomitantly. All in all, the children evaluated 18 samples containing emotional speech: 9 noisified (3 only audio, 3 audio + positive drawing, and 3 audio + negative drawing) and 9 clear (same distribution as before). The two samples that contain only visual stimuli, that is, the emotionally connoted drawings, have been evaluated with the purpose of guaranteeing their intended emotional connotation.

In order to promote interest and to avoid fatigue, the stimuli were divided into five short tasks, each presenting four randomly selected stimuli out of the total 20, lasting between 2 and 3 min. The tasks (whose order could be chosen by the child) had different-coloured interfaces and could be performed by the children consecutively or in separate sessions, depending on their interest. All participants gave positive feedback and performed voluntarily the tasks in one session which lasted around 10 to 15 min. For each stimulus, the caregiver instructed the children to listen carefully (wearing headphones) and to observe the image on the screen (when visual stimuli were considered). Then, the children were asked about the emotional state of the speaker, and invited to select on their own one of the three emotional faces. An informal interview was performed at the end by the caregiver in which the children were asked about the goal of the task in order to ensure that they understood it, by that guaranteeing the validity of the responses.

A total of 109 children took part in the perception test (cf. Table 1). 92 children were Spanish: 17 (6 male, 11 female) from the *preoperational* stage (4–6 years); 53 (34 male, 19 female) from the *concrete operational* stage (7–10 years); and 22 (8 male, 14 female) from the *formal operational* stage (11–14 years). 17 children were German (6 male, 11 female) from the *preoperational* stage (4–6 years).^5^ All the children performed the test in their native language. Out of the 92 Spanish children, 62 were recruited via the collaboration of a public Spanish primary school (*CPI Plurilingüe Cabo da Area* of Laxe), the remaining 30 via the voluntary participation of their parents. The 17 German children were recruited via the collaboration of a kindergarten. The setting with a familiar caregiver leading the test (teacher or parent) guaranteed a natural behaviour.^6^ In order to evaluate how differences in the perception of emotional speech may have a relationship with adults’ assessment, the perception test was also performed by 17 Spanish adults (12 male, 5 female), with ages from 17 to 48 years (mean 31, standard deviation 7.5). The adults were recruited directly by the experimenters and were middle class native Spanish from the same region as the Spanish children (i. e., Galicia).

**Table 1:** Distribution of the 109 children considering: age (*preoperational*: stage 4–6 years; *concrete operational* stage: 7–10 years; *formal operational* stage: 11–14 years), gender (male and female), and nationality (Spanish and German)

Age	#	Spanish		German	
		Male	Female	Male	Female
Preop.					
4	10	–	5	1	4
5	16	5	3	2	6
6	8	1	3	3	1
Total	34	6	11	6	11
Concrete					
7	12	12	–	–	–
8	16	8	8	–	–
9	24	14	10	–	–
10	1	–	1	–	–
Total	53	34	19	–	–
Formal					
11	13	4	9	–	–
12	4	1	3	–	–
13	1	–	1	–	–
14	4	3	1	–	–
Total	22	8	14	–	–

## Statistical design

Our statistical analysis is based on a two-level hierarchical structure of the data: at Level-1, we define repeated measures nested within participants, that is, the user variables; at Level-2, observations relate to the experimental setup, that is, the task variables. For Level-1, we consider four independent variables (i)–(iv) and a dependent (v) one: (i) user-id (nominal: 34 categories for the cross-cultural assessment, 109 for the general assessment, 34 for adults vs. children assessment); (ii) age (scale: from 4 to 6 years for the cross-cultural assessment, from 4 to 14 for the general assessment; binary: *kid* and *adult* for adults vs. children assessment); (iii) gender (binary: *female* and *male*); (iv) nationality (binary: *Spanish* and *German*); (v) perception (binary: *correct* and *incorrect*). For Level-2, we consider four independent (i)–(iv) variables: (i) task-id (nominal: 18 categories from question 1 to question 18); (ii) snr (binary: *clean* and *noisy*); (iii) reinforcement (nominal: *encouraging*, *discouraging*, and *none*); (iv) emotion (nominal: *happiness*, *anger*, and *sadness*). In Table 2, a summary of the variables considered in the statistical analysis is given. We assume encouraging reinforcement when the emotion displayed in the drawing matches the emotional content of the speech, discouraging when there is no match, that is, the visual stimulus contradicts the emotional content of the speech, and no reinforcement when no visual stimulus is given.^7^

**Table 2:** Summary of variables considering: hierarchical structure (user variables at Level-1 and task variables at Level-2), type, measurement, and values

Variable	Level	Type	Measurement	Values
user-id	1—user var.	Independent	Nominal	Cross-cultural: participant 1–34
			Nominal	General: participant 1–109
			Nominal	Adult/children: participant 1–34
age	1—user var.	Independent	Scale	Cross-cultural: 4–6 years
			Scale	General: 4–14 years
			Binary	Adult/children: *adult*, *child*
gender	1—user var.	Independent	Binary	*Female*, *male*
nationality	1—user var.	Independent	Binary	*Spanish*, *German*
perception	1—user var.	Dependent	Binary	*Correct*, *incorrect*
task-id	2—task var.	Independent	Nominal	*Question* 1–18
snr	2—task var.	Independent	Binary	*Noisy*, *clean*
reinforcement	2—task var.	Independent	Nominal	*Positive*, *negative*, *none*
emotion	2—task var.	Independent	Nominal	*Sadness*, *anger*, *happiness*

Note that for user-id and age, different values are considered for each assessment: cross-cultural, general, and adults vs. children)

We employ Generalised Linear Mixed Models (GLMM) from SPSS (Corporation 2012). Robust estimation of fixed effect and covariances, as well as binary logistic regression, to relate the target distribution to the model (due to the dependent variable perception being binary), were considered. Given the unbalanced sample size between Spanish and German children (92 vs. 17), and between the adults’ control group and the total of involved children (17 vs. 109), we divided the statistical evaluation in three subsections: the cross-cultural assessment (only children from 4 to 6 years, both Spanish and German, were considered, i. e., 34 children in total), general assessment (all the children, both Spanish and German, were considered, i. e., 109 children in total), and adults versus children’s assessment (only Spanish children from 4 to 6 years and the adults were considered, i. e., 34 individuals in total). In order to estimate the variability of the responses across participants, that is, whether it might be any difference in performing the perception task across individuals, an unconditional (null) logistic regression model without predictors—no independent variables were included—was performed as starting configuration of the model for the three assessments (cross-cultural, general, and adults vs. children). Considering that the individual observations are nested simultaneously within the task-id and the user-id variables (all the participants answer all the questions), we approach the repeated measures nature of our data by considering these two variables as crossed random effects (Baayen et al. 2008). In addition, as covariance type for the random effects, Variance Component (VC)—the default setting option in SPSS—was considered to build up the model. Finally, in the data structure, the variable perception was indicated as a target, the variables task-id and user-id as subjects. When the *z*-test of the unconditional model indicated intercept variance, thus suggesting that the independent variables might influence participants’ responses, multilevel evaluation was performed by hierarchically adding into the model the independent variables: first those of Level-1, then those of Level-2; note that the variables that did not play a role were subsequently excluded (Heck et al. 2013). Since the variables reinforcement and emotion have three different classes, dummy coding was employed to evaluate them: as a reference, no reinforcement, and happiness (the only positive emotion) were considered.

The following statistical parameters are employed: We evaluate the magnitude and direction of the effects shown by the model in terms of odds ratios; yet, since odds ratios might be poorly understood in the communication of research findings (Grant 2014), we additionally convert them into the more common effect size measure Cohen’s *d* (Borenstein et al. 2009). For the (null) model, we report in the text *z*-test and *p*-values. However, Null-Hypothesis-Testing with *p*-values as decisive criterion has been critised repeatedly from its beginning; we refer to the statement of the American Statistical Association in Wasserstein and Lazar (2016). Throughout this article, we will thus report *p*-values not as criteria for a binary ‘significant/not significant’ decision but rather as a descriptive device; note that we do not correct the *p*-values for repeated measurements. For the model with Level-1 and Level-2 variables, we discuss Cohen’s *d* in the text; F-statistic, degrees of freedom, coefficient $\beta$, exp(B), Cohen’s *d*, *p*-values, and 95% confidence intervals, are always given in the tables. For reporting statistical results, two floating points are used except for the *p*-values where the usual three are displayed. Percentages are indicated with no floating points since most of the time, the number of evaluated instances was less than 100. For reproducibility, we make the SPSS syntax and dataset considered for the present study freely accessible.^8^

## Results

### Cross-cultural assessment

**Table 3:** Results for the fixed effects computed in the cross-cultural assessment considering Level-1 variables

Factor	F	df1	df2	$\beta$	exp(*B*)	*d*	*p*	95% CI	
								Lower	Upper
age	2.44	1	608	0.24	1.28	0.14	.119	0.94	1.74
gender	0.11	1	608	− 0.08	0.92	− 0.05	.739	0.58	1.47
nationality	0.04	1	608	− 0.04	0.96	− 0.02	.842	0.63	1.46

A Generalized Linear Mixed Model (GLMM) was performed on the responses given by 17 Spanish and 17 German children from the *preoperational* stage (4–6 years), considering task-id and user-id as crossed random effects, age, gender, and nationality as fixed effects; F-statistic (*F*), degrees of freedom 1 (df1) and 2 (df2), Coefficient $\beta$, effect sizes *exp(B)* and Cohen’s *d*, *p*–value, and 95% confidence intervals (CI): lower and upper, are given

It has been shown that culture influences a child’s perception of emotional speech, that is, children from different cultures show different sensitivity toward emotions (McCluskey and Albas 1981; Matsumoto and Kishimoto 1983). In order to evaluate whether there is a difference between Spanish and German children in performing the presented task, a GLMM was employed on the data collected from the *preoperational* stage (4–6 years): 34 children (12 male, 22 female), 17 Spanish and 17 German (cf. Table 1). The unconditional (null) logistic regression model without predictors shows variability in intercepts across the different questions, as indicated by the *z*-test for task-id ($z=2.12$, $p=.034$), which suggests that the independent variables might influence the children’s responses, thus encouraging the development of a multilevel evaluation. Still, the model without predictors did not show variability in intercepts across the different children, as indicated by the *z*-test for user-id ($z=1.53$, $p=.126$), which is probably due to the low number of subjects. Despite this, in order to evaluate whether the variables within Level-1, that is age, gender, and nationality, might explain the variability across tasks, we defined them as fixed effects—note that perception is kept in the model as target, task-id and user-id are kept as crossed random effects.

Table 3 displays the results for the model considering Level-1 variables as fixed effects, which estimated that the correlation between age and the probability of choosing the right answer is positive, that is, the likelihood of older children to correctly identify emotional speech is higher than for younger; still, the effect size indicates that this tendency is small ($d=0.14$). Regarding gender, we observe that for females, the probability to answer correctly decreases. However, keeping age and nationality constant, the reduced likelihood of a female to properly identify emotions with respect to a male is negligible, as indicated by the very small effect size ($d = -\,0.05$). Similar results hold for nationality, for which our model estimated that being Spanish slightly decreases the probability to answer correctly with respect to German; still, this difference is also minimal, as shown by an even smaller effect size ($d = -\,0.02$). The *z*-test showed similar results as previously: $z=2.13$, $p=.033$ for task-id and $z=1.45$, $p=.147$ for user-id; yet, given the small effect sizes displayed by the fixed effects, and due to the risk of model overspecification (given the small number of participants), no further evaluation was made with the cross-cultural subset of the data.

### General assessment

**Table 4:** Results for the fixed effects computed in the general assessment considering Level-1 variables

Factor	F	df1	df2	$\beta$	exp(*B*)	*d*	*p*	95% CI	
								Lower	Upper
age	6.80	1	1959	0.07	1.08	0.04	.009	1.02	1.14
gender	0.15	1	1959	0.06	1.06	0.03	.695	0.80	1.41

The GLMM was performed on the responses given by the 109 children, considering task-id and user-id as crossed random effects, age and gender as fixed effects; F-statistic (F), degrees of freedom 1 (df1) and 2 (df2), coefficient $\beta$, effect size: *exp(B)* and Cohen’s *d*, *p*–value, and 95% confidence intervals (CI): lower and upper, are given

To find out whether the probability for the 109 children to correctly perceive emotional speech varies across tasks, again the unconditional (null) logistic regression model without predictors was performed—considering this time all the children, both Spanish and German, from 4 to 14 years (i. e., all the participants in Table 1). Our analysis shows that there is intercept variance across users between the different questions: $z=2.50$, $p=.012$ for task-id and $z=4.12$, $p=.000$ for user-id; this encourages a multilevel evaluation. To evaluate whether Level-1 variables, that is, age and gender, might explain this variability, these two variables were added into the model as fixed effects. As previously, perception was kept in the model as target, task-id and user-id as crossed random effects—meaning that the evaluation of fixed effects and further random effects will be performed by adding these elements to the current configuration of the model.

Table 4 displays the results for the model considering Level-1 variables as fixed effects, which again indicates that age is positively correlated with the probability to properly identify emotional speech: keeping gender constant, older children increase their likelihood to give the right response—notice that even though the effect size is very small ($d=0.04$), this increment is per year, and for age ten levels have been considered. Regarding gender, we observe that for females, the probability to answer correctly slightly increases, but as well as in the cross-cultural assessment, the difference between genders is minimal, as shown by a very small effect size ($d=0.03$). Once again, the *z*-test suggests multilevel interactions: $z=2.50$, $p=.012$ for task-id and $z=3.98$, $p=.000$ for user-id; this was evaluated by adding the Level-2 variables into the model.

Table 5 displays the results for the model with Level-1 and Level-2 variables, which was performed in order to evaluate whether the relationship between age and probability to answer correctly might be linked to the task variables. For this, we specified age (user variable) as random effect and added snr, reinforcement, and emotion (task variables) as fixed effects—gender, since not relevant, was no longer considered in the equation (Heck et al. 2013). The model estimated that snr influences the correlation between age and the likelihood to correctly identify emotions in speech, that is, the probability of children to answer correctly increases in clean conditions, which is shown by a medium effect size ($d=0.47$). By evaluating Level-2 variables, reinforcement did not influence children’s responses, as displayed by the very small effect sizes for both positive ($d = 0.01$) and negative ($d = -\,0.05$) reinforcement. emotion showed a slightly bigger (although small) effect size, indicating that children are more likely to answer correctly when evaluating sadness ($d = 0.24$) than when assessing anger ($d = 0.17$). By looking at the random effects, the z-test did not encourage further evaluation, displaying $z = 0.91$, $p =.362$ for task-id and $z = 0.21$, $p =.837$ for the level-1 slope; $z = 0.51$, $p =.614$ for user-id and $z = 1.64$, $p =.101$ for the level-1 slope.

**Table 5:** Results for the fixed effects computed in the general assessment considering Level-1 and Level-2 variables

Factor	F	df1	df2	$\beta$	exp(*B*)	*d*	*p*	95% CI	
								Lower	Upper
snr	31.28	1	1955	0.85	2.34	0.47	.000	1.74	3.76
reinf (*positive*)	0.01	1	1955	0.02	1.02	0.01	.920	0.71	1.47
reinf (*negative*)	0.24	1	1955	− 0.09	0.91	− 0.05	.628	0.63	1.32
emo (*sadness*)	5.43	1	1955	0.44	1.55	0.24	.020	1.07	2.23
emo (*anger*)	2.56	1	1955	0.30	1.35	0.17	.110	0.94	1.94
age	3.91	1	1955	0.06	1.06	0.03	.048	1.00	1.12

The GLMM was performed on the responses given by the 109 children, considering task-id and user-id as crossed random effects, the Level-1 slope age as randomly varying, and the Level-2 predictors: snr, reinforcement–reinf (positive and negative w. r. t. the reference no reinforcement), emotion–emo (the negative emotions sadness and anger w. r. t. the positive emotion happiness), and the Level-1 predictor age as fixed effects; F-statistic (F), degrees of freedom 1 (df1) and 2 (df2), coefficient $\beta$, effect size: *exp(B)* and Cohen’s *d*, *p*–value, and 95% confidence intervals (CI): lower and upper, are given

### Adults versus children’s assessment

To perform a balanced comparison and to avoid cultural bias, the 17 Spanish adults’ responses and those of the 17 Spanish children from the *Preoperation* stage (4–6 years) were evaluated. Again, to examine the variability of the responses from the 34 participants across the different questions, the unconditional (null) logistic regression model without predictors was performed by taking into account the 17 adults and the 17 children. The model shows again variability in intercepts across the different questions ($z=2.06$, $p=.040$ for task-id) and children ($z=2.15$, $p=.032$ for user-id), thus encouraging multilevel evaluation. To evaluate whether Level-1 variables, that is, age and gender, might explain this variability, these two variables were added into the model as fixed effects; unlike previously, age was considered as a binary variable: adults (17–48 years) versus children (4–6 years). Again, from now on, perception was kept in the model as a target, task-id and user-id as crossed random effects.

**Table 6:** Results for the fixed effects computed in the adults versus children’s assessment considering Level-1 variables

Factor	F	df1	df2	$\beta$	exp(*B*)	*d*	*p*	95% CI	
								Lower	Upper
age	20.52	1	609	1.02	2.80	0.57	.000	1.79	4.38
gender	0.74	1	609	0.19	1.21	0.11	.391	0.78	1.89

GLMM was performed on the responses given by the 17 Spanish children from the *preoperational* stage (4–6 years) and the 17 Spanish adults (17–48 years), considering as crossed random effects task-id and user-id; as fixed effects age and gender; F-statistic (F), degrees of freedom 1 (df1) and 2 (df2), coefficient $\beta$, effect size: *exp(B)* and Cohen’s *d*, *p*–value, and 95% confidence intervals (CI): lower and upper, are given

Table 6 displays the results for the model considering Level-1 variables as fixed effects. As expected, the model predicted that the correlation between age and the likelihood to properly identify an emotion is positive, which indicates that, holding gender constant, the likelihood of adults to answer correctly increases, which is shown by a medium-large effect size ($d=0.57$). As in the general assessment, gender seems not to play a role in perception of emotional speech, as shown by the very small effect size ($d=0.11$), indicating that the differences between females and males are minimal. The model showed variability in intercepts across the different questions ($z=2.18$, $p=.038$ for task-id) but not between participants ($z=1.07$, $p=.284$ for user-id), which is probably due, as previously indicated in the cross-cultural assessment, to the low number of subjects. Despite this, again, in order to evaluate multilevel interactions, Level-2 variables were added into the model.

**Table 7:** Results for the fixed effects computed in the adults versus children’s assessment considering Level-1 and Level-2 variables

Factor	F	df1	df2	$\beta$	exp(*B*)	*d*	*p*	95% CI	
								Lower	Upper
snr	14.42	1	605	0.90	2.45	0.49	.000	1.54	3.90
reinf (*positive*)	0.03	1	605	0.05	1.05	0.03	.886	0.60	1.84
reinf (*negative*)	0.00	1	605	0.01	1.01	0.01	.976	0.57	1.78
emo (*sadness*)	9.63	1	605	0.89	2.45	0.49	.002	1.39	4.31
emo (*anger*)	7.76	1	605	0.79	2.20	0.43	.006	1.26	3.84
age	14.64	1	605	1.01	2.74	0.56	.000	1.63	4.59

GLMM was performed on the responses given by the 17 Spanish children from the *preoperational* stage (4–6 years) and the 17 Spanish adults (17–48 years), considering task-id and user-id crossed random effects; the Level-1 slope age (binary: child vs. adult) randomly varying; the Level-2 predictors: snr, reinforcement–reinf (positive and negative w. r. t. the reference no reinforcement), emotion–emo (the negative emotions sadness and anger w. r. t. the positive emotion happiness), and the Level-1 predictor age as fixed effects; F-statistic (F), degrees of freedom 1 (df1) and 2 (df2), coefficient $\beta$, effect size: *exp(B)* and Cohen’s *d*, *p*–value, and 95% confidence intervals (CI): lower and upper, are given

Table 7 displays the results for the model with Level-1 and Level-2 variables, which was performed in order to examine if the relationship between age (user variable) and the probability to give a correct response might be linked to the variables snr, reinforcement, and emotion (task variables). age was added into the model as random effect, and snr, reinforcement, and emotion as fixed effects, while gender, since irrelevant, was not considered (Heck et al. 2013). The model estimated that snr and emotion are influential factors in the correlation between age and the likelihood to answer correctly, that is, keeping age constant, the probability to correctly identify emotions increases in clean conditions ($d=0.49$), as well as when evaluating negative emotions, a phenomenon that is slightly more prominent for sadness ($d=0.49$) than for anger ($d=0.43$). This confirms previous findings (Parada-Cabaleiro et al. 2018), suggesting that both arousal and valence are relevant dimensions for children in the evaluation of emotional speech—thus, sadness (low aroused and negative) is identified with more accuracy than happiness (high aroused and positive), with anger (high aroused and negative) in between. reinforcement was again not relevant, which is shown by a very small effect size for both positive ($d = 0.03$) and negative ($d = 0.01$) reinforcements. These findings, however, should be carefully interpreted, since the model lead to an overspecification warning, compromising the computation of z-test. This is due, as previously indicated, to the relatively high number of stimuli (18), considering the limited number of users (34).

## Discussion

**Fig. 4:**
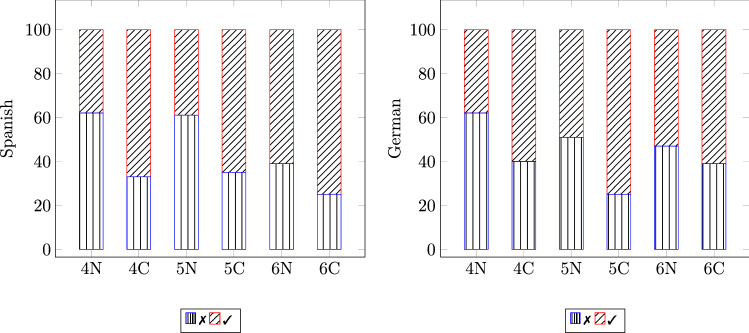
Percentage of wrong (✗) and correct (✓) responses in the identification of the emotional speech in both snr: noisy (N) and clean (C), by Spanish and German children of the Preoperational Stage (4–6 years)

**Fig. 5:**
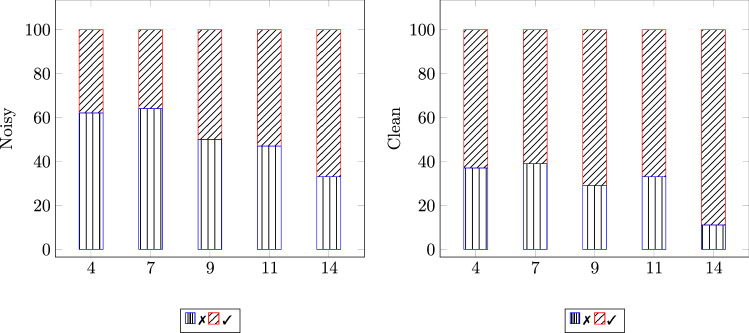
Percentage of wrong (✗) and correct (✓) responses in the identification of the emotional speech in both snr (noisy and clean), by children of 4, 7, 9, 11, and 14 years—notice that for children of 4 years both Spanish and German children are considered together

Our statistical analysis shows that the user variables at Level-1, gender and nationality, do not influence listeners’ perception of emotional speech, whereas the variable age is positively correlated with the probability to answer correctly; thus, showing that the ability to identify emotions in speech develops with age. Against our expectations, the presented study also indicates that the Task variable at Level-2, visual reinforcement, does not influence the perception of emotion—neither for children nor for adults; yet, other visual stimuli should also be investigated in order to generalise such finding. As expected, the variable snr influences the perception of emotional speech for both children and adults, regardless of culture. This can be seen in Fig. 4, which shows that in clean conditions, the percentage of correct responses is always higher than that of incorrect ones for both cultural groups (Spanish and German) across the three evaluated ages (4–6 years). In noisy background, however, the percentage of wrong answers is higher than that of correct ones, for both children of 4 and 5 years, while only for the oldest children (6 years), the percentage of correct responses is slightly higher than that of incorrect ones. This suggests that the difficulty of identifying emotions in adverse environmental conditions decreases with age—a tendency that is displayed when evaluating the remaining age groups. To illustrate such a tendency, some representative age groups— excluding groups with a very low number of participants—were considered, that is, 4, 7, 9, 11, and 14 years. In Fig. 5 it is displayed that in clean conditions, across the evaluated ages, the percentage of utterances correctly identified is higher than the one of incorrect ones. In noisy background, however, the percentage of wrong answers is higher than the one for correct answers; this tendency is inverted after the age of 9 years. This is shown by the Unweighted Average Recall^9^ (UAR) as well, which in background noise progressively increases with age, demonstrating that the impairing effect of adverse environmental conditions decreases with age (cf. UAR for Noise in Table 8). There is a weak, positive correlation between children’s age and UAR (2-tailed, Pearson: $r=.252$, $p<.008$, Spearman: ${\rho }=.245$, $p<.010$) for samples with background noise. This correlation is markedly lower for clean samples (2-tailed, Pearson: $r=.165$, $p<.087$, Spearman: ${\rho }=.156$, $p<.106$). This indicates that younger children might have developed the ability to correctly identify emotions in speech; yet, this can easily be impaired by adverse conditions such as background noise.

**Table 8:**
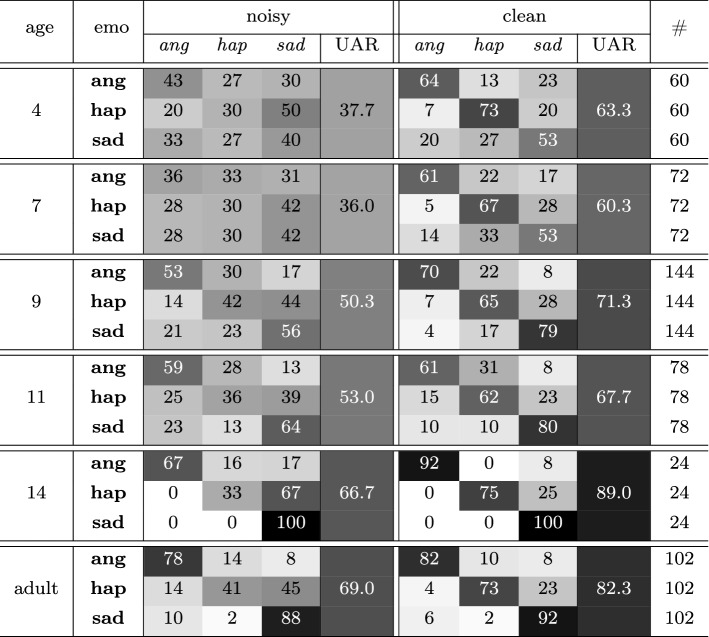
Confusion matrix for the percentage of accuracy in the perception of each emotion (emo): anger (ang), happiness (hap), and sadness (sad); by children of 4, 7, 9, 11, and 14 years (cf. Table 1) and adults (17–48 years); in both snr (noisy and clean)

In each row, the reference is given (emotions indicated in bold); in each column, ‘identified as’ is given (emotions indicated in italics). Darker shadowing represents higher levels of accuracy; Unweighted Average Recall (UAR) and number of responses encoded in each row (#) are given as well

When evaluating the confusion patterns between emotions, we observe that in background noise, happiness is the emotion worst identified, displaying similar accuracy across the different age groups (e. g., 30% and 33% for children of 4 and 14 years, respectively), whereas the correct identification of anger and sadness progressively increases with age (cf. confusion matrices for noisy in Table 8). Indeed, even though the perception of emotional speech by the older children (14 years) in clean conditions is comparable to that performed by adults, differences between these two age groups are observed when evaluating the confusion patterns displayed in background noise—children identify anger and happiness worse than adults: 14 years old children achieve an accuracy of $67\%$ and $33\%$ for anger and happiness (cf. noisy for age 14 in Table 8); adults achieve an accuracy of $78\%$ and $41\%$ for anger and happiness (cf. noisy for adult in Table 8). This suggests that children in the last stage of the developmental age might have already acquired the capacity to fully identify these emotions in speech; still, this is an ability that has not been refined to the same level as in adults, and might be more easily impaired in adverse environmental conditions. Note that the higher performance of 14 years old children for some cells (cf. Table 8) might be also explained by the difference in sample size: 4 children versus 17 adults.

Supporting previous research (Parada-Cabaleiro et al. 2017), we can see a most prominent one-directed confusion: happiness is misidentified as sadness. This is particularly evident in noisy background regardless age, as displayed for both adults and children: the percentage of happiness wrongly perceived as sadness is higher than for those cases correctly identified (for instance, $50\%$ vs. $30\%$ and $45\%$ vs. $41\%$ for happiness misclassified vs. correctly identified for 4 years old children and adults, respectively; cf. Table 8). Indeed, in background noise, the acoustic properties of emotional speech are attenuated—pitch and energy are obfuscated, less discernible, and therefore, it is more difficult to tell apart them from the characteristics of sadness. By acoustically evaluating F0 and energy ranges^10^ of each emotional utterance, we see that anger shows the highest differences in range (F0 range $= 433.09$ Hz, energy range $= 0.13$), sadness the lowest (F0 range $= 159.56$ Hz, energy range $= 0.08$), and happiness is in between (F0 range $= 256.33$ Hz, energy range $= 0.12$); cf. Fig. 1. This explains that in background noise anger, being more prominent than happiness, is better recognised (for instance, with an accuracy of $43\%$ in 4 years old children, cf. Table 8). On the other side, since sadness is ‘typically’ an acoustically less prominent emotion, the confusion between this emotion and happiness shows up mostly in one direction, that is, happiness is misclassified as sadness, but sadness is not misclassified as happiness. This phenomenon is clearly shown for adults’ perception in background noise: $41\%$ versus $45\%$ for happiness correctly identified versus misclassified and $88\%$ versus $2\%$ for sadness correctly identified versus misclassified (cf. Table 8).

## Limitations of the presented study

Even though we put much effort in developing a methodology which is adequate for our age groups, the extent to which the lower performance observed for younger children might be due to cognitive difficulties in the understanding of the task and not—or to a lesser extent—to poorer emotional skills, cannot be answered unequivocally. In this regard, we firmly believe that the performance of the presented task in a familiar environment is essential to maximise the validity of the results. This is shown, for example, by the children’s curiosity about the meaning of the sentences and the language of the speaker—demonstrating, as expected, that the children perceived the nonsensical utterances as a foreign language. The fact that visual reinforcement did not influence the perception of emotional speech does not necessarily mean that emotionally contradictory visual stimulation does not play a role, but instead it might also relate to our procedure being not sufficiently immersive. Note that with our work, we present a first attempt to evaluate the influences of visual stimuli—implicitly related to an emotional content—in the perception of emotional speech; this differs with previous research (Shackman and Pollak 2005) where the visual stimuli associated to the emotional cues were facial expressions, that is socially accepted icons that explicitly represent emotional expressions. Considering the outcomes presented by Shackman and Pollak (2005) which show that a contradictory visual reinforcement influences children’s perception of emotional speech, our findings might indicate that the implicit stimuli used by us were too abstract to influence children’s perception; for a deeper understanding of the topic, further evaluation should be done by taking into account a larger selection of explicit and implicit emotionally-connoted visual stimuli.

Our results are consistent with the scant literature on the development of cognitive and affective empathy, and on the development of the social brain (Theory of Mind, ToM), from childhood to adolescence. Richardson et al. (2018) report “evidence that ToM and pain networks are functionally distinct by 3 years of age, and become increasingly specialized between the ages of 3–12 years” (p. 3). The result of Sánchez-Pérez et al. (2014) are in line with other research findings “that both cognitive and affective empathy increased with age [i. e., 9 to 18 years], although the effect sizes were small” (p. 2). Moreover, our dilemma that with our experimental design, we cannot really tell apart cognitive and affective aspects, is shared by practically all experimental approaches: We do not know whether the tasks in the lab are processed by our subjects the same way as the tasks in real life they want to model.

Other aspects are the unbalanced sample size and the limited cultural diversity. The unequal distribution of children across age groups might not only have biased the results to a certain extent but has also impaired the evaluation of gender related patterns within each age group. This is a frequent problem in recruiting subjects from groups (such as classes at schools or universities) with inherently unbalanced distributions; we took this into account by conducting separate analyses, that is, the cross-cultural, general, and children vs. adult assessments. The consideration of two specific cultures impedes the generalisation of our conclusions, as they, strictly speaking, only hold for the groups considered. Indeed, since previous findings have shown that even children from different European countries may present diverse emotional intelligence skills (Lahaye et al. 2011), further evaluation is still needed not only by comparing highly dissimilar cultures, as, for example, Asian and European, but also within European countries.

## Conclusions

In this work, we assessed the extent to which background noise and multi-modal stimuli influence children’s perception of emotional speech throughout different stages of the cognitive development. The visual reinforcement employed did not have an effect, whereas artificially superimposed noise significantly decreased the ability to identify emotions in speech correctly. The influence of noise decreased with age, and it affected the perception of happiness most predominately. This is most likely due to happiness being an emotion acoustically less characterised than anger and sadness: Happiness shows medium levels of F0 and energy range, whereas anger and sadness show ‘extreme’ levels (anger the highest, sadness the lowest). We found no differences between children from the two evaluated cultures (German and Spanish)—this might be due to the similarity between both cultures. Differences in the confusion patterns displayed in the perception of emotional speech in background noise between adults and older children suggest that the full development of such skills is only achieved in higher age.

In future work, we will consider children coming from more diverse cultures, in order to evaluate if adverse conditions and multi-modal stimuli may influence the perception of emotional speech differently, depending on a child’s cultural background. Our goal with this and future work is to develop an understanding of children’s perception of emotional speech, and by that, to contribute to the advancement of educational, psychological, and technological areas of research, for example, to develop artificially intelligent systems based on child–computer-interaction for psycho-pedagogic purposes (Song et al. 2019).

## Footnotes

1 Realised in GEMEP as the sub-category ‘elation’.

2 Realised in GEMEP as the sub-category ‘hot anger’.

3 https://www.typeform.com/.

4 These two stimuli were the emotionally connoted drawings mentioned above, which were evaluated in the same perception test as the other stimuli.

5 Different from the definition of the stages given in Sect. 2, the years of each stage are disjunct, for obvious reasons.

6 Consent to use the anonymised responses of each child for research purposes only was obtained through caregivers’ signed consent, and under the approval of the TUM IRB ethics committee (Hantke et al. 2016).

7 Note that Reinforcement might also be considered as an ordinal variable: for instance, 1 (negative), 2 (none), and 3 (positive); yet, since we want to consider ‘no reinforcement’ as a reference for the dummy coding, we have chosen to identify this variable as a nominal.

8 https://amzn.to/2KQFEGV.

9 Unweighted Average Recall is the unweighted (by number of instances in each class) mean of percent correctly classified in the diagonal of the confusion matrix. Chance level is only defined by the number of classes (50 % for two classes, 33.3 % for three classes, and so on) and not by the number of cases per class which varies across experiments (Schuller and Batliner 2014).

10 Both features (F0 range and energy range) were extracted from the ComParE feature set (Schuller et al. 2013) by the openSMILE feature extractor (Eyben et al. 2010).

## References

[CR1] Baayen RH, Davidson DJ, Bates DM (2008). Mixed-effects modeling with crossed random effects for subjects and items. Journal of Memory and Language.

[CR2] Bänziger T, Scherer KR, Scherer KR, Bänziger T, Roesch EB (2010). Introducing the Geneva multimodal emotion portrayal (GEMEP) corpus. Blueprint for affective computing: A sourcebook.

[CR3] Bänziger, T., Pirker, H., & Scherer, K. (2006). GEMEP-GEneva multimodal emotion portrayals: A corpus for the study of multimodal emotional expressions. In: Proceedings of the LREC’06 Workshop on Corpora for Research on Emotion and Affect, ELRA, Genova, Italy, pp. 15–19.

[CR4] Batliner, A., Schuller, B., Schaeffler, S., & Steidl, S. (2008). Mothers, adults, children, pets: Towards the acoustics of intimacy. In: Proceedings of the International conference on acoustics, speech and signal processing, IEEE, Las Vegas, NV, pp. 4497–4500.

[CR5] Bent T, Holt RF (2018). Shhh... I need quiet! Children’s understanding of American, British, and Japanese-accented English speakers. Language and Speech.

[CR6] Borenstein M, Hedges LV, Higgins J, Rothstein HR (2009). Introduction to meta-analysis.

[CR7] Corporation IBM (2012). IBM SPSS statistics for windows, version 21.0.

[CR8] Darwin C (1872). The expression of the emotions in man and animals.

[CR9] Eyben, F., Wöllmer, M., & Schuller, B. (2010). Opensmile: The Munich versatile and fast open-source audio feature extractor. In: Proceedings of the ACM Multimedia, ACM, Florence, pp. 1459–1462.

[CR10] Finkelstein, S.L., Nickel, A., Harrison, L., Suma, E.A., & Barnes, T. (2009). cMotion: A new game design to teach emotion recognition and programming logic to children using virtual humans. In: Proceedings of the Virtual reality conference, IEEE, Lafayette, LA, pp. 249–250.

[CR11] Friend M, Bryant JB (2000). A developmental lexical bias in the interpretation of discrepant messages. Merrill-Palmer Quarterly.

[CR12] Friend M, Farrar MJ (1994). A comparison of content-masking procedures for obtaining judgments of discrete affective states. The Journal of the Acoustical Society of America.

[CR13] Fritschi L, Brown A, Kim R, Schwela D, Kephalopoulos S (2011). Burden of disease from environmental noise: Quantification of healthy life years lost in Europe.

[CR14] Grant RL (2014). Converting an odds ratio to a range of plausible relative risks for better communication of research findings. British Medical Journal.

[CR15] Gumenyuk V, Korzyukov O, Alho K, Escera C, Schröger E, Ilmoniemi RJ, Nätänen R (2001). Brain activity index of distractibility in normal school-age children. Neuroscience Letters.

[CR16] Hantke S, Weninger F, Kurle R, Ringeval F, Batliner A, Mousa AED, Schuller B (2016). I hear you eat and speak: Automatic recognition of eating condition and food type, use-cases, and impact on asr performance. PLoS ONE.

[CR17] Heck RH, Thomas S, Tabata L (2013). Multilevel modeling of categorical outcomes using IBM SPSS.

[CR18] House D (2009). On the perception of mood in speech: Implications for the hearing impaired. Lund Working Papers in Linguistics.

[CR19] Ives SW (1984). The development of expressivity in drawing. British Journal of Educational Psychology.

[CR20] Klorer PG (2009). The effects of technological overload on children: An art therapist’s perspective. Art Therapy.

[CR21] Lahaye M, Mikolajczak M, Rieffe C, Villanueva L, Van Broeck N, Bodart E, Luminet O (2011). Cross-validation of the emotion awareness questionnaire for children in three populations. Journal of Psychoeducational Assessment.

[CR22] Mathworks, Inc. (2014). *MATLAB: R2014a*. Natick, MA: Mathworks, Inc.

[CR23] Matsumoto D, Kishimoto H (1983). Developmental characteristics in judgments of emotion from nonverbal vocal cues. International Journal of Intercultural Relations.

[CR24] McCluskey KW, Albas DC (1981). Perception of the emotional content of speech by canadian and mexican children, adolescents, and adults. International Journal of Psychology.

[CR25] Misailidi P, Bonoti F (2008). Emotion in children’s art: Do young children understand the emotions expressed in other children’s drawings?. Journal of Early Childhood Research.

[CR26] Morton JB, Trehub SE (2001). Children’s understanding of emotion in speech. Child Development.

[CR27] Morton JB, Trehub SE, Zelazo PD (2003). Sources of inflexibility in 6-year-olds’ understanding of emotion in speech. Child Development.

[CR28] Most T, Michaelis H (2012). Auditory, visual, and auditory–visual perceptions of emotions by young children with hearing loss versus children with normal hearing. Journal of Speech, Language, and Hearing Research.

[CR29] Öster AM, Risberg A (1986). The identification of the mood of a speaker by hearing impaired listeners. Speech Transmission Laboratory Quarterly Progress and Status Reports.

[CR30] Parada-Cabaleiro, E., Baird, A., Batliner, A., Cummins, N., Hantke, S., & Schuller, B. (2017). The perception of emotions in noisified non-sense speech. In: Proceedings of the Interspeech, Annual Conference of the International Speech Cassociation, ISCA, Stockholm, pp. 3246–3250.

[CR31] Parada-Cabaleiro, E., Costantini, G., Batliner, A., Baird, A., & Schuller, B. (2018). Categorical vs dimensional perception of Italian emotional speech. In: Proceedings of the Interspeech, Annual Conference of the International Speech Communication Association, ISCA, Hyderabad, pp. 3638–3642.

[CR32] Piaget J (1962). The relation of affectivity to intelligence in the mental development of the child. Bulletin of the Menninger Clinic.

[CR33] Piaget J, Lee K (2000). Piaget’s theory. Childhood cognitive development: The essential readings.

[CR34] Quam C, Swingley D (2012). Development in children’s interpretation of pitch cues to emotions. Child Development.

[CR35] Richardson H, Lisandrelli G, Riobueno-Naylor A, Saxe R (2018). Development of the social brain from age three to twelve years. Nature Communications.

[CR36] Russell JA (1980). A circumplex model of affect. Journal of Personality and Social Psychology.

[CR37] Sánchez-Pérez N, Fuentes LJ, Jolliffe D, González-Salinas C (2014). Assessing children’s empathy through a Spanish adaptation of the basic empathy scale: Parent’s and child’s report forms. Frontiers in Psychology.

[CR38] Scharenborg, O., Kakouros, S., Koemans, J., et al. (2018). The effect of noise on emotion perception in an unknown language. In: Proceedings of the International Conference on Speech Prosody, pp. 364–368.

[CR39] Scherer KR, Scherer KR, Ekman P (1984). Expression and the nature of emotion. Approaches to emotion.

[CR40] Scherer KR, Banse R, Wallbott HG (2001). Emotion inferences from vocal expression correlate across languages and cultures. Journal of Cross-cultural Psychology.

[CR41] Schuller B, Batliner A (2014). Computational paralinguistics: Emotion, affect and personality in speech and language processing.

[CR42] Schuller, B., Steidl, S., Batliner, A., Vinciarelli, A., Scherer, K., Ringeval, F., Chetouani, M., Weninger, F., Eyben, F., Marchi, E., Mortillaro, M., Salamin, H., Polychroniou, A., Valente, F., & Kim, S. (2013). The Interspeech 2013 computational paralinguistics challenge: Social signals, conflict, emotion, autism. In: Proceedings of the Interspeech, Annual Conference of the International Speech Communication Association, ISCA, Lyon, pp. 148–152.

[CR43] Shackman JE, Pollak SD (2005). Experiential influences on multimodal perception of emotion. Child Development.

[CR44] Song, M., Yang, Z., Baird, A., Parada-Cabaleiro, E., Zhang, Z., Zhao, Z., & Schuller, B. (2019). Audiovisual analysis for recognising frustration during game-play: Introducing the multimodal game frustration database. In: Proceedings of the ACII, International Conference on Affective Computing and Intelligent Interaction, IEEE, Cambridge, pp. 517–523.

[CR45] Wasserstein RL, Lazar NA (2016). The ASA’s statement on p-values: Context, process, and purpose. The American Statistician.

[CR46] Waxer M, Morton JB (2011). Children’s judgments of emotion from conflicting cues in speech: Why 6-year-olds are so inflexible. Child Development.

[CR47] Winston AS, Kenyon B, Stewardson J, Lepine T (1995). Children’s sensitivity to expression of emotion in drawings. Visual Arts Research.

